# Association of Genetic Variation in Adaptor Protein APPL1/APPL2 Loci with Non-Alcoholic Fatty Liver Disease

**DOI:** 10.1371/journal.pone.0071391

**Published:** 2013-08-19

**Authors:** Michelangela Barbieri, Antonietta Esposito, Edith Angellotti, Maria Rosaria Rizzo, Raffaele Marfella, Giuseppe Paolisso

**Affiliations:** Department of Medical, Surgical, Neurological, Metabolic and Geriatric Sciences, Second University of Naples, Naples, Italy; University of Tor Vergata, Italy

## Abstract

The importance of genetics and epigenetic changes in the pathogenesis of non alcoholic fatty liver disease (NAFLD) has been increasingly recognized. Adiponectin has a central role in regulating glucose and lipid metabolism and controlling inflammation in insulin-sensitive tissues and low adiponectin levels have been linked to NAFLD. *APPL1* and *APPL2* are adaptor proteins that interact with the intracellular region of adiponectin receptors and mediate adiponectin signaling and its effects on metabolism. The aim of our study was the evaluation of a potential association between variants at *APPL1* and *APPL2* loci and NAFLD occurrence. The impact on liver damage and hepatic steatosis severity has been also evaluated. To this aim allele frequency and genotype distribution of *APPL1*- rs3806622 and -rs4640525 and *APPL2*-rs 11112412 variants were evaluated in 223 subjects with clinical diagnosis of NAFLD and compared with 231 healthy subjects. The impact of *APPL1* and *APPL2* SNPs on liver damage and hepatic steatosis severity has been also evaluated. The minor-allele combination *APPL1-*C/*APPL2*-A was associated with an increased risk of NAFLD (OR = 2.50 95% CI 1.45–4.32; p<0.001) even after adjustment for age, sex, body mass index, insulin resistance (HOMA-IR), triglycerides and adiponectin levels. This allele combination carrier had higher plasma alanine aminotransferase levels (Diff = 15.08 [7.60–22.57] p = 0.001) and an increased frequency of severe steatosis compared to the reference allele combination (OR = 3.88; 95% CI 1.582–9.531; p<0.001). In conclusion, C-*APPL1*/A-*APPL2* allele combination is associated with NAFLD occurrence, with a more severe hepatic steatosis grade and with a reduced adiponectin cytoprotective effect on liver.

## Introduction

Nonalcoholic fatty liver disease (NAFLD) is the most common chronic liver disease in the Western population, encompassing a spectrum of histological injury ranging from pure benign steatosis to progressive necro inflammation and fibrosis [1]–[2]. Altered adipokine action and increased oxidative stress are candidate pathogenetic mechanisms in NAFLD [3]–[6]. Among the different adipokines, low adiponectin levels predict severity of liver disease in NAFLD, even in the absence of diabetes and obesity [5]–[6]. Adiponectin exerts its effects through two membrane receptors, AdipoR1 and AdipoR2 [7]. Although the post receptor signalling remains to be elucidated, it seems that adiponectin achieves its function in the liver via activating 5-AMP-activated protein kinase (AMPK) and peroxisome proliferator-activated receptor (PPAR)-α pathways [3]–[4], [8]. Adaptor protein, PH domain and leucine zipper containing 1 (APPL1) and Adaptor protein, PH domain and leucine zipper containing 2 (APPL2) are the first identified adaptor proteins that interact directly with adiponectin receptors [9]–[10]. They are both highly expressed in insulin target tissues, including skeletal muscle, liver and adipose tissue and mediate adiponectin signaling and its effects on metabolism via binding to N terminus of adiponectin receptors [9]–[10]. Adiponectin signaling through APPL1 is necessary to exert its anti-inflammatory and cytoprotective effects on endothelial cells [11]–[12]. APPL1 also functions in insulin-signaling pathway [13] and is an important mediator of adiponectin dependent insulin sensitization in skeletal muscle and also acts as a mediator of other signaling pathways by interacting directly with membrane receptors or signalling proteins, thereby playing critical roles in cell proliferation, apoptosis, cell survival, endosomal trafficking, and chromatin remodelling [14]. The function of APPL2 is not entirely clear even if it was reported to down regulate the ADIPO R1 signaling [15]. Interestingly, over expression of APPL1 in mouse hepatocyte cells can activate p38 mitogen-activated protein kinases (MAPK), and adiponectin treatment could further enhance this effect, suggesting that APPL1 plays a role in adiponectin signaling in liver [9]. Genetic as well as environmental factors are important in the development of NAFLD. While it is well known that environmental risk factors influence the development and progression of NAFLD [16]–[17], the contribution of the individual genetic variation to disease predisposition remains uncertain, despite the fact that several genes, including PPARα, phosphatidyl ethanolamine methyltransferase (PEMT), and Patatin like phospholipase-3 (PNPLA3), have been suggested as potential candidates for either NAFLD susceptibility or disease progression [18]–[21]. Since APPL1 and APPL2 mediate the effects of adiponectin on target tissues, they have been considered to be strong candidates in the pathogenesis of NAFLD. Thus, we hypothesized that genetic variants in APPL1 and APPL2 genes might affect the NAFLD risk through the effects on adiponectin signaling.

Common single-nucleotide polymorphisms (SNPs) have been identified in both APPL1 and APPL2 gene. Previous studies found a significant association between an APPL1 gene variant with body fat distribution in Type 2 Diabetes Mellitus (T2DM) [22] although no association with development of prediabetes phenotypes [23] in a healthy metabolically well-characterized population has been demonstrated. A significant association of APPL2 genetic variation with overweight and obesity has been also found [24]. To our knowledge, the contribution of these SNPs to liver lipid accumulation and liver function has not yet been investigated.

The aim of our study will be the evaluation of a potential association between the APPL1 and APPL2 loci and the occurrence and progression of non alcoholic fatty liver disease (NAFLD). To this aim, allele frequency and genotype distribution of rs3806622 and rs4640525 of APPL1 and rs 11112412 of APPL2 gene variants were evaluated in subjects with clinical diagnosis of NAFLD and compared with control healthy subjects. The impact of APPL1 and APPL2 SNPs on liver damage and hepatic steatosis severity has been also evaluated.

The APPL1- rs3806622 and -rs4640525 and *APPL2*-rs 11112412 variants were chosen on the basis of their high MAF (0. 435, 0.489, 0.193) and previous evidences showing their significant association with body fat and its distribution and with cardiovascular risk in type 2 diabetes [22], [24]–[25]. Other variants have been previously studied and found in linkage disequilibrium with them.

## Materials and Methods

223 Caucasians subjects (129 males and 94 females; mean age: 55±11 years) with clinical diagnosis of NAFLD, living in southern Italy and referred to our Department, were enrolled. The diagnosis of NAFLD was established based on the following inclusion criteria: (1) persistently abnormal levels of aspartate aminotransferase and alanine aminotransferase (AST/ALT), (2) evidence of fatty liver using ultrasound imaging techniques, and (3) screened negative for viral markers (such as HBsAg, anti-HBc, anti-HBs, anti-HCV, HCV RNA and anti-HIV) [26]–[29].

Additionally, 231 healthy individuals (114 males and 117 females; mean age: 54±14 years), matched for sex, age, body mass index (BMI), ethnicity and life style, without steatosis by ultrasonography, free of any known major diseases were also enrolled.

Patients with the following diseases were excluded from the study: infectious hepatitis (hepatitis B and C, Epstein-Barr virus infection), autoimmune hepatitis, primary biliary cirrhosis, sclerosing cholangitis, hemochromatosis, α1-antitrypsin deficiency, Wilson’s disease, drug-induced hepatitis, alcoholic hepatitis, and excessive alcohol consumption (present or past daily consumption of more than 20 g alcohol per day). Patients affected by diabetes were also excluded. Clinical information was obtained by routine laboratory analysis, history and physical examination. The maximal alcohol consumption of the study participants was 30 g per week in men and 20g in woman.

### Ethics Statement

The use of human blood sample and the protocol in this study were strictly conformed to the principles expressed in the Declaration of Helsinki and were approved by the Institutional Ethical Committees of the Second University of Naples. Written informed consent was obtained from all participants before their participation in the study.

### Liver Ecography

Non-invasive evaluation of the distribution pattern of liver fatty infiltration was performed by ecography [27]. The echography patterns of fatty liver disease appears as “bright” liver (brightness and posterior attenuation) with stronger echoes in the hepatic parenchyma than in the renal parenchyma, vessel blurring and narrowing of the lumens of the epatic veins in the absence of findings, which are suggestive of other cronic liver desease. Mild, moderate or severe steatosis degree was defined according to the following items [27]: 1. Diffuse enhancement of near field echo in the hepatic region (stronger than in the kidney and spleen region) and gradual attenuation of the far field echo. 2. Unclear display of intra-hepatic lacuna structure. 3. Mild to moderate hepatomegaly with a round and blunt border. 4. Color Doppler ultrasonography shows a reduction of the blood flow signal in the liver or it is even hard to display, but the distribution of blood flow is normal. 5. Unclear or non-intact display of envelop of right liver lobe and diaphragm.

Mild degree of fatty liver displays item 1 and any one of items 2–4; moderate fatty liver displays item 1 and any two items of items 2–4; severe fatty liver displays items 1 and 5 and any two of items 2–4 [27].

### Analytical Methods

Anthropometric determinations (weight, height, BMI, and waist/hip ratio) were measured by standard techniques. Plasma fasting AST, ALT, gamma glutamil transferasi (G-GT), total cholesterol, LDL cholesterol, HDL cholesterol, triglycerides, ferritin, glucose, adiponectin, insulin, were determinated by routine laboratory methods (Roche Diagnostics, Monza (MI), Italy).

### Genetic Polymorphism

All individuals were genotyped for rs3806622 and rs4640525 polymorphisms of the APPL1 gene on chromosome 3p21.1-p14.3 and for rs11112412 polymorphism of the APPL2 gene on chromosome 12q 24.1. Genomic DNA was obtained from blood lymphocytes collected into EDTA-containing tubes using a commercial DNA extraction kit (Illustra, GE Healthcare UK Limited, Buckinghamshire, HP7 9 NA,UK). Reference APPL1 and APPL2 genome sequence were obtained from the NCBI database (Homo Sapiens Chromosome, GeneBank AB037849 and AY113704). Primers chosen for amplification were designed using Primer 3 software. Amplification was performed using the following primers: APPL1-rs4640525-F: 5′-TTTGTGGAATTGGTCAGGTG-3′; APPL1- rs4640525-R: 5′-AGCAAGATCCCCATCTCAAA-3′) APPL1-rs3806622-F (5′-TGGATTTGTTCCCATGTATCTG-3′; APPL1-rs3806622-R: 5′-TGAGGGCT ACAAG CCTATCCT-3′) and APPL2- rs11112412-F (5′-ATTCAACAAGGGCACAGTCC -3′) APPL2- rs11112412-R (5′- CATTGCCAGCGAGTGTTCTA-3′).

The Polymerase chain reaction (PCR) was carried out under the following conditions: 95°C for 5 min, followed by 35 cycles of 95°C for 30 s, 60°C for 30 s, 72°C for 1 min, with final extension of 72°C for 4 min. Genotyping was performed using restriction analysis (RFLP) using the *Hpy188I* enzyme for APPL1 rs4640525, the *BfaI* enzyme for APPL1 rs3806622 and *Hind III* for APPL2 rs11112412 and incubating at 37°C over night. The resulting products were identified on 4.5% agarose gel.

Laboratory personnel who assessed all genotyping results were blinded to the samples case–control status. Genotyping quality was examined by a detailed quality control procedure consisting of 95% successful call rate, duplicate calling of genotypes, internal positive control samples and Hardy–Weinberg Equilibrium (HWE) testing. The concordance rate was greater than 99% based on 10% of duplicate samples for each SNP.

Directly DNA sequencing was use in a subset of 50 individuals to further confirm the genotypes for each SNP.

### Statistical Analysis

Insulin resistance (HOMA-IR) was calculated according to the homeostasis model assessment (HOMA) (30–31): insulin resistance (IR) = FI x G/22.5 where FI = fasting insulin (mU/ml) and G = fasting glucose (mmol/l) [30]–[31].

For each SNP, the genotypic and allelic frequencies were calculated. The Chi square test was used to compare the expected genotypic frequencies with the actual frequencies observed based on the Hardy–Weinberg equilibrium. The difference in genotype frequency between NAFLD and controls was analyzed by standard contingency Chi square test. Univariate and multivariate logistic regression analyses were performed to obtain the crude and adjusted odds ratios (ORs) for risk of NAFLD and their 95% confidence intervals (CIs). Age, sex, HOMA (IR), plasma adiponectin and triglycerides levels and BMI were considered as potential confounders and were also included in the multivariate logistic regression models.

Differences between groups were analyzed by ANOVA when variables were normally distributed; otherwise, the Mann–Whitney U-test was used. The Bonferroni correction was applied to the analysis of study groups. Associations between genotypes and metabolic profile parameters were analyzed using the General Linear Model.

Statistical analyses were performed using SPSS software package. All metabolic parameters are presented as means ± standard deviation (SD). In order to investigate differences between the two study groups, sample size was estimated by GPOWER software. The resulting sample size, estimated according to a global effect size of 0.25 with type I error of 0.05 and a power of 95% was 210 patients.

Linkage disequilibrium and allele combination analyses were performed by use of the Thesias program based on the stochastic-EM algorithm [32]. The Thesias program allows estimation of both haplotype frequencies and covariable-adjusted haplotype effects by comparison with a reference haplotype taken as the most frequent haplotype in the current analyses. A global test of association between haplotypes and any studied phenotype was performed by means of a chi-square test with m-1 degree of freedom in the case of m haplotypes.

## Results

Clinical and biochemical characteristics of the study subjects are reported in Table 1. All subjects (n = 454) were adult (mean age = 54.9±13), overweight, with no difference in gender ratio (211 F/243 M, χ2 = 2.25 p = 0.130). Compared to control subjects, NAFLD patients had higher occurrence of most of the risk factors of the metabolic syndrome, including elevated blood pressure, IR (HOMA) index, fasting plasma glucose, total cholesterol, triglycerides and decreased HDL-C. In addition, levels of alanine transaminase (ALT) and aspartate transaminase (AST) were significantly higher in patients with NAFLD, compared to controls. No difference in gender distribution was found in both groups (Table1).

**Table 1:** Clinical characteristics of NAFLD patients and control subjects.

	Control group	p	NAFLD
	(n = 231)		(n = 223)
Age (years)	54±14	.985	55±11
Gender (M/F)	114/117		129/94
BMI (kg/m^2^)	29.2±1.8	.361	29.4±1.8
Glucose (mg/dl)	78±20	.001	92±18
Total Cholesterol (mgl/dl)	188±36	.025	201±41
HDL Cholesterol (mg/dl)	60±16	.001	50±14
Triglycerides (mmol/l)	86±36	.001	142±121
AST (U/L)	20.1±10	.001	51.0±24
ALT (U/L)	23.9±15	.001	102±23
Adiponectin (µg/ml)	12.3±6.7	.001	7.4±4.4
HOMA index	2.54±1.17	.012	3.84±1.41
Ferritin (ng/ml)	486±224	.003	59±28
Systolic Blood Pressure (mmHg)	124±5	.005	146±8
Diastolic Blood Pressure (mmHg)	75±3	.003	88±6

### Genetic Analysis

In all study groups, the genotype frequencies of all gene variants studied respected the Hardy-Weinberg equilibrium (p>0.05 for all the SNP investigated). The genotypic frequencies observed for all gene variants studied were almost similar to those reported in previous studies among Caucasians.

A strong linkage disequilibrium between the *rs4640525* and *rs3806622* APPL1 gene polymorphisms studied was observed (D′ = 0.97, r^2^ = 0.81). Since these two variants would be predicted to provide identical/nearly identical genotypic information the rs3806622 variant was excluded from further analyses.

The minor allele frequencies was 0.456 for *rs4640525* APPL1 gene polymorphisms and 0.184 for *rs11112412* APPL2. The frequency of APPL1 rs4640525 GG, GC and CC genotypes was 30.4%, 48% and 21.6% respectively. As far as *rs11112412* APPL2 gene variant is concerned the frequency of GG, GA and AA genotypes was 66.3%, 30.6% and 3.1.% respectively.

### Association of APPL1 and APPL2 Genes with NAFLD

Genotype distributions of APPL1 and APPL2 polymorphisms in patients with NAFLD and control subjects and their associations with NAFLD are summarized in Table 2. A different rs 4640525 APPL1 genotype distribution was found between NAFLD and healthy control subjects while no differences were found when genotype distribution of APPL2 gene polymorphism was analyzed. Indeed, both the C allele at the APPL1 locus and the A allele at the APPL2 locus were found to be more represented among NAFLD subjects in comparison to controls.

**Table 2:** Genotype and allele frequencies distribution of rs4640525- APPL1 and rs11112412-APPL2 in NAFLD patients and control.

	CONTROL	NAFLD	*Odds ratio (95%CI)*			
			*crude*	*p*	*Adjusted* *	*P*
**APPL1**						
GG	37.7 (87)	22.9(51)	1		1	
GC	44.6 (103)	51.6(115)	2.02 (1.24–3.27)	.004	1.901 (1.23–2.94)	.123
CC	17.7 (41)	25.6 (57)	2.90 (1.60–5.26)	.001	2.372 (1.39–4.02)	.001
χ^2^ = 12.5; p = 0.002						
*Allele Frequency*						
*g*	60(277)	48.7 (217)	1		1	
*c*	40(185)	51.3 (229)	1.73 (1.28–2.32)	.002	1.56(1.20–2.03)	.0001
Fisher’s test: *P = *0.001						
**APPL2**						
GG	71.0 (164)	61.4 (137)	1		1	
GA	26.4(61)	35.0 (78)	1.80 (1.14–2.80)	.001	1.531 (1.02–2.29)	.030
AA	2.6 (6)	3.6 (8)	1.79 (0.54–5.90)	.331	1.590 (0.54–4.71)	.397
χ^ 2^ = 4.64; p = 0.098						
*Allele Frequency*						
*g*	84.2 (389)	78.9(352)	1		1	
*a*	15.8(73)	21.1 (94)	1.635(1.11–2.41)	.010	1.436(1.02–2.02)	.003
Fisher’s test: *P* _ 0.04						

Data are presented as % (n);

* adjusted for age, BMI, IR HOMA index, plasma adiponectin and triglycerides levels.

A logistic regression analysis was used to estimate associations between the APPL1 and APPL2 variant and the risk of NAFLD (Table 2). It was found that individuals with at least one copy of the rs4640525 C allele and rs11112412 APPL2 A allele had respectively a 1.73 (95% CI: 1.28–2.32; P = 0.002) and 1.63 (95% CI: 1.11–2.41; P = 0.01) fold increased risk compared with those without this allele. Adjustment for age, BMI, HOMA IR, triglycerides and adiponectin levels did not appreciably change the association.

Considering the possibility of interchromosomal interaction, allele combination analysis of APPL1 and APPL2 variants have been performed. Allele combination frequencies in NAFLD and control subjects are shown in Table 3. When the most common allele combination consisting of the 2 major alleles G-G considered as reference, as expected the all-minor-allele combination CA was associated with an increased risk of NAFLD (OD = 2.50 95% CI 1.45–4.32; p<0.0009) even after adjustment for age, sex, BMI, HOMA IR, triglycerides and adiponectin levels.

**Table 3:** Allele combination effect on NAFLD risk.

Allele Combination		Frequency					
APPL1	APPL2	CONTROL	NAFLD	*Odds ratio (95%CI)*	p	*Adj Odds ratio (95%CI)* *	p
G	G	0.54	0.44	1.0		1.0	
G	A	0.05	0.04	1.182 (0.50–2.76)	.700	0.915 (0.39–2.10)	.834
C	G	0.30	0.34	1.564(1.11–2.19)	.009	1.392 (1.02–1.89)	.033
C	A	0.09	0.16	2.50 (1.45–4.32)	.001	2.152 (1.38–3.34)	.0001

* adjusted for age, BMI, IR HOMA index, plasma adiponectin and triglycerides levels.

### Impact on Quantitative Traits

**Figure 1:**
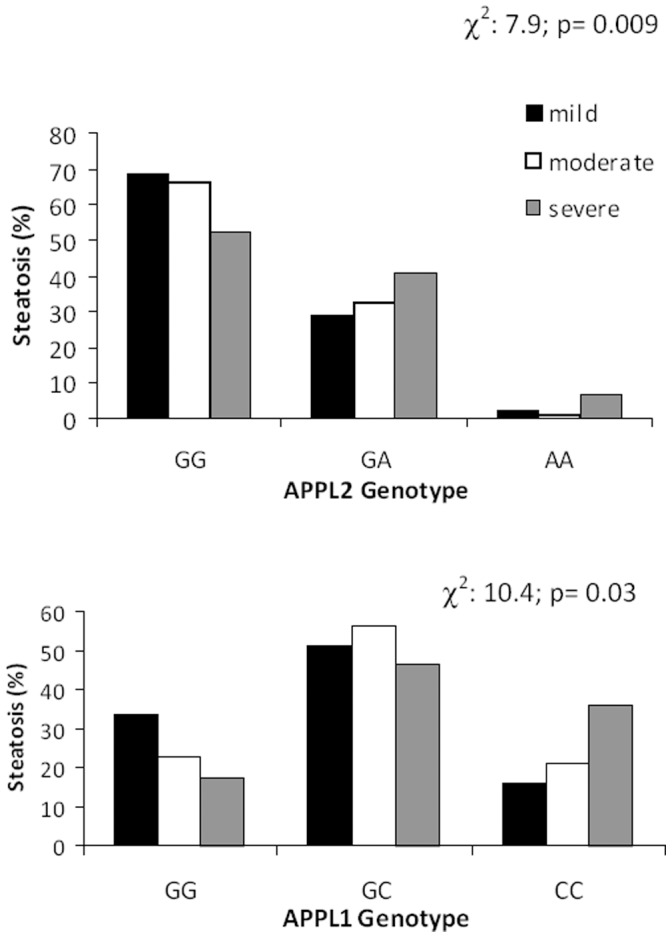
Hepatic steatosis severity among APPL1 and APPL2 genotype groups.

The impact of genetic variability at APPL1 and APPL2 on AST and ALT plasma levels was tested in univariate analysis (Table 4). APPL1 and APPL2 genotypes affected ALT plasma levels, whereas no difference in AST plasma levels according to genotypes was observed. Accordingly, the effect of APPL1 and APPL2 polymorphisms on ALT levels was assessed by means of a General Linear Model ANOVA, including age, IR (HOMA) and BMI, plasma triglycerides and adiponectin levels as covariates.The analysis revealed higher plasma ALT levels in *C*+subjects (GC and CC genotypes) in comparison to C- subjects (GG genotype): 59.48±1.89 vs 46.02±2.87 U/L, F = 15.2, p = 0.003, as well as in APPL2 A+ individuals (GA and AA genotypes) in comparison to A- individuals (GG genotypes): 61.39±2.74 vs 52.36±1.95, F = 7.12, p = 0.008). Comparing the different grading of hepatic steatosis among the three genotype groups there was an increased frequency of severe steatosis in NAFLD individuals with APPL1-CC genotype; a trend for individuals with APPL2 AA genotype to have an higher frequency of severe steatosis was also found; the differences however did not reach statistical significance (Figure 1).

**Table 4:** Levels of alanine transaminase (ALT) and aspartate transaminase (AST) among different APPL1 and APPL2 genotypes.

	AST	ALT
**APPL1- rs4640525**		
GG	31.7±16.3	45.4±33.0
GC	34.9±16.5	59.0±37.4*
CC	37.0±18.8	61.2±36.2*
C+	31.7±16	59.7±37 **^§^**
C-	35.5±17.3	45.4±33
**APPL2 - rs11112412**		
GG	33.7±16.5	52.6±35.3
GA	35.9±18.2	60±37
AA	34.8±18.0	65±44
A+	35.8±18.15	60.8±38.1 **^§^**
A-	33.7±16	52.6±35.0

* p<0.05 vs GG genotype; § p<0.05 vs No carrier.

A logistic regression analysis revealed that individuals with at least one copy of the rs4640525 C allele and rs11112412 APPL2 A allele had a 2.17 (95% CI: 1.27–3.711; P = 0.004) and 1.96 (95% CI: 1.0008–3.83; P = 0.01) fold increased risk for severe steatosis respectively.

Further allele combination-phenotype analyses revealed a significant association of CA allele combination with ALT plasma levels and grading of hepatic steatosis after adjustment for age, BMI, HOMA IR, adiponectin and triglycerides plasma levels. This allele combination carriers had higher plasma ALT levels (Diff = 15.08679 [7.60247–22.57111] p = 0.000078) and an increased frequency of severe steatosis compared to the reference allele combination (Table 5).

**Table 5:** Allele combination effect on plasma ALT levels and hepatic steatosis severity.

Allele Combination			*Steatosis frequency*			*Odds ratio 95%CI)for Severe steatosis*	p	*Expected plasma ALT levels odds ratio (95%CI )* *		p	
APPL 1	APPL2	Mild		Moderate	Severe						
G	G	0.52		0.46	0.33	1.0		23.95 [20.95–26.96]			
G	A	0.07		0.04	0.07	1.27 [0.43–3.74]	.660	21.45 [7.04–35.85] Diff = −2.50 [−17.5–12.55]	.744		
C	G	0.32		0.35	0.39	1.92 [1.05–3.48]	.030	29.70[25.84–33.56] Diff = 5.74[0.24–11.23]	.040		
C	A	0.09		0.13	0.19	3.88 [1.58–9.53]	.003	39.04 [32.4–45.63662] Diff = 15.08[7.60–22.57]	.0001		

* Phenotypic Mean = 55.39; Standard Error = 36.46; Residual Standard Error = 35.74.

## Discussion

In the present study, we investigated the effect of two SNPs at APPL1 and APPL2 loci in relation to NAFLD occurrence. The major finding of this study is that the rs4640525-APPL1/rs11112412-APPL2 CA allele combination is associated with an increased occurrence of NAFLD after controlling for potential confounders, such as age, BMI, HOMA index and adiponectin plasma levels. The analysis also revealed higher plasma ALT levels and a more severe hepatic steatosis grade for CA allele combination carriers.

NAFLD is recognized as the most common type of chronic liver disease in Western countries and the leading cause of cryptogenic cirrhosis [1]–[2]. The importance of genetic and epigenetic changes in the aetiology and pathogenesis of NAFLD has been increasingly recognized and a large number of SNPs related to NAFLD has been documented by candidate gene studies [18]–[21], [33] To our knowledge, this is the first study providing evidence of an association between APPL1 and APPL2 genetic variants and NAFLD in humans.

Although the pathogenesis of NAFLD is not fully elucidated, a complex interaction between adipokines and cytokines produced by adipocytes and/or inflammatory cells infiltrating adipose tissue appears to play a crucial role in Metabolic Syndrome and NAFLD [3]–[6].

Adiponectin is the most abundant and adipose-specific adipokine. There is an evidence that adiponectin decreases hepatic and systematic IR and attenuates liver inflammation and fibrosis. Insulin resistance (IR) is a key factor in the pathogenesis of NAFLD, the latter being considered as the hepatic component of IR or metabolic syndrome [4], [34]. The insulin-sensitizing effect of adiponectin appears to be mainly through enhancement of fatty acid oxidation and glucose uptake in muscle and inhibition of gluconeogenesis in liver [7]–[8], which are mediated by two cell surface receptors, AdipoR1 and AdipoR2 [7]–[8], by a direct interaction with the extracellular COOH terminus of these receptors [7], [9].

APPL1 and APPL2 have been suggested as the “missing link” in the adiponectin-signaling cascade, transmitting signals from adiponectin receptors to downstream targets by directly interacting with the NH_2_-terminal intracellular region of AdipoR1 and AdipoR2 [9].

Adiponectin signaling through APPL1 is necessary to exert its anti-inflammatory and cytoprotective effects on endothelial cells [9]–[14]. APPL1 also functions in insulin-signaling pathway and is an important mediator of adiponectin dependent insulin sensitization in skeletal muscle [9]–[14]. Furthermore, over expression of APPL1 in mouse hepatocyte cells activate p38 MAPK, and adiponectin treatment further enhance this effect, suggesting that APPL1 plays a role in adiponectin signaling in liver [10]. The function of APPL2 is not entirely clear even if recently, it has also been suggested to play an important role in adiponectin and insulin signaling as well as the cross-talk between these two pathways [15].

Previous studies have demonstrated a significant association between APPL1 gene variants and body fat distribution in T2DM [22] and a significant association of APPL2 genetic variation with overweight and obesity [24]. The association of APPL1 and APPL2 variant with NAFLD, hepatic function and distribution pattern of liver fatty infiltration has not been previously explored. Our results, firstly demonstrate an association of rs4640525APPL1 G/C and rs11112412 -APPL2 GA variants with NAFLD being the A allele at the APPL2 locus and the C allele at the APPL1 locus more represented among NAFLD subjects in comparison to controls. Moreover, the CA allele combination was associated with a 2.5 fold increased risk of NAFLD even after adjustment for age, sex, BMI, HOMA IR and adiponectin levels.

The mechanism through which genetic variants at APPL1 or APPL2 locus could influence NAFLD occurrence can not been confirmed. It is likely that the SNPs investigated, which are placed in intronic regions, are only genetic markers in strong LD with other genetic variants having biological effects. Indeed, we can hypothesize that the SNPs studied may differently affect the expression levels of APPL1 or APPL2, which leads to an altered adiponectin activity, a decrease in AMPK activation, hepatic glucose uptake and free fatty acid (FFAs) oxidation and an increase in de novo lipogenesis and gluconeogenesis, resulting in intrahepatic lipid accumulation and fatty liver. The final effect is an impaired adiponectin cytoprotective effects on liver. In agreement, in our study both higher rs4640525-C and rs11112412-A NAFLD risk alleles have been found associated with higher plasma ALT levels and a more severe hepatic steatosis grade.The effect found was independent of plasma adiponectin level underlining that, despite normal or high adiponectin levels, an impaired post receptor signalling due to APPL1/APPL2 SNPS may alter adiponectin efficiency and activity. Accordingly, in cultured myotubes, APPL1 knockdown lowers adiponectin- stimulated fatty acid oxidation, glucose uptake, and phosphorylation of AMPK, acetyl-CoA carboxylase (ACC) and p38 [9]. Likewise, overexpression of wild-type APPL1 enhances basal glucose transporter type 4 (GLUT4) glucose transporter type 4translocation, while overexpression of dominant negative APPL1 inhibits basal, insulin- and adiponectin stimulated glucose trasporter type 4 (GLUT4) translocation [35].

Interestingly, the significant interaction found between APPL1 and APPL2 strongly support the hypothesis that interaction with a different genetic and/or environmental background may differently modulate the effect of a given gene in different populations. APPL2 is an isoform of APPL1 and can form a dimer with APPL1 [36]–[37]. APPL2 can negatively regulate adiponectin signaling by competing with APPL1 in binding to AdipoR1 [15]. Indeed, suppression of APPL2 can promote adiponectin-stimulated glucose uptake and fatty acid oxidation [15].Although, unlike APPL1, APPL2 does not directly interact with the catalytic subunit of PI3-kinase and Akt2 which are key kinases in the PI3-kinase pathway downstream of the insulin receptor [36]–[37], APPL2 can suppress insulin signalling by inhibiting the interaction between APPL1 and the components of insulin signaling. Thus, suppression of APPL2 greatly enhanced the sensitizer effect of adiponectin on insulin [15].

Other SNPs of genes regulating insulin signalling, lipid metabolism, oxidative stress, inflammation have previously associated with NAFDL, including APOC3, PNPLA3, FABP1, TNFa, HF3, PEMT, but the contribution of the individual genetic variation to diseases predisposition remains uncertain [38]. Although there is evidence that these genetic factors account for considerable variability in susceptibility to NAFLD, most studies have not been well validated by larger replication cohorts. Only PNPLA3 gene variant has been more extensively examined and validated by a genome wide association study [38].

In conclusion, our study demonstrates that C-APPL1/A-APPL2 allele combination is associated with an increase NAFLD occurrence, with a more severe hepatic steatosis grade and with a reduced adiponectin cytoprotective effects on liver. Such an effect is probably due to the combined effect of APPL1 and APPL2 genes on both adiponectin and insulin signaling.

Potential limitations in our study need to be addressed. Firstly, the diagnosis of NAFLD was primarily based on ultrasonographic findings. Indeed for ethical reasons, it is very difficult to perform liver biopsies in an epidemiological survey. Secondly, our study firstly reported the relationship between SNPs of APPL1 and APPL2 genes and NAFLD in an Italian population subjects. Thus, further studies will be necessary for replicating our finding in an independent larger population group and other races.

## References

[1] Farrell GC, Larter CZ (2006) Nonalcoholic fatty liver disease: from steatosis to cirrhosis. Hepatology 43: S99–S112. 16447287 10.1002/hep.20973

[2] Bedogni G, Miglioli L, Masutti F, Tiribelli C, Marchesini G, et al (2005) Prevalence of and risk factors for nonalcoholic fatty liver disease: the Dionysos nutrition and liver study. Hepatology 42: 44–52. 15895401 10.1002/hep.20734

[3] Polyzos SA, Kountouras J, Zavos C, Tsiaousi E (2010) The role of adiponectin in the pathogenesis and treatment of non-alcoholic fatty liver disease. Diabetes Obes Metab 12(5): 365–83. 20415685 10.1111/j.1463-1326.2009.01176.x

[4] Polyzos SA, Kountouras J, Zavos C (2009) Non alcoholic fatty liver disease: the pathogenetic roles of insulin resistance and adipocytokines. Curr Mol Med 72: 299–314. 10.2174/156652409787847191 19355912

[5] Musso G, Gambino R, Durazzo M, Biroli G, Carello M, et al (2005) Adipokines in NASH: postprandial lipid metabolism as a link between adiponectin and liver disease. Hepathology 42: 1175–1183. 10.1002/hep.20896 16231364

[6] Targher G, Bertolini L, Rodella S, Zoppini G, Scala L, et al (2006) Associations between plasma adiponectin concentrations and liver histology in patients with nonalcoholic fatty liver disease. Clin Endocrinol (Oxf) 64(6): 679–83. 16712671 10.1111/j.1365-2265.2006.02527.x

[7] Yamauchi T, Kamon J, Ito Y, Tsuchida A, Yokomizo T, et al (2003) Cloning of adiponectin receptors that mediate antidiabetic metabolic effects. Nature 423: 762–769. 12802337 10.1038/nature01705

[8] Yamauchi T, Kamon J, Minokoshi Y, Ito Y, Waki H, et al (2002) Adiponectin stimulates glucose utilization and fatty-acid oxidation by activating AMP-activated protein kinase. Nat Med 8: 1288–1295. 12368907 10.1038/nm788

[9] Mao X, Kikani CK, Riojas RA, Langlais P, Wang L, et al (2006) APPL1 binds to adiponectin receptors and mediates adiponectin signalling and function. Nat Cell Biol 8: 516–523. 16622416 10.1038/ncb1404

[10] Deepa SS, Dong LQ (2009) APPL1: role in adiponectin signaling and beyond. Am J Physiol Endocrinol Metab 296(1): E22–E36. 18854421 10.1152/ajpendo.90731.2008 PMC2636986

[11] Wang Y, Cheng KK, Lam KS, Wu D, Wang Y, et al (2011) APPL1 counteracts obesity-induced vascular insulin resistance and endothelial dysfunction by modulating the endothelial production of nitric oxide and endothelin-1 in mice. Diabetes 60(11): 3044–54. 21926268 10.2337/db11-0666 PMC3198090

[12] Cheng KK, Lam KS, Wang Y, Huang Y, Carling D, et al (2007) Adiponectin-induced endothelial nitric oxide synthase activation and nitric oxide production are mediated by APPL1 in endothelial cells. Diabetes 56: 1387–1394. 17287464 10.2337/db06-1580

[13] Cheng KK, Iglesias MA, Lam KS, Wang Y, Sweeney G, et al (2009) APPL1 potentiates insulin- mediated inhibition of hepatic glucose production and alleviates diabetes via Akt activation in mice. Cell Metab 9: 417–42. 19416712 10.1016/j.cmet.2009.03.013

[14] Hosch SE, Olefsky JM, Kim JJ (2006) APPLied mechanics: uncovering how adiponectin modulates insulin action. Cell Metab 4(1): 5–6. 16814726 10.1016/j.cmet.2006.06.003

[15] Wang C, Xin X, Xiang R, Ramos FJ, Liu M, et al (2009) Yin-Yang regulation of adiponectin signaling by APPL isoforms in muscle cells. J Biol Chem 284(46): 31608–31615. 19661063 10.1074/jbc.M109.010355 PMC2797231

[16] Suzuki A, Lindor K, St Saver J, Lymp J, Mendes F, et al.. (2005) Effect of changes on body weight and lifestyle in non-alcoholic fatty liver disease. J Hepatol 43, 1060–1066. 10.1016/j.jhep.2005.06.008 16140415

[17] Speliotes EK, Yerges-Armstrong LM, Wu J, Hernaez R, Kim LJ, et al (2011) Genome-wide association analysis identifies variants associated with non-alcoholic fatty liver disease that have distinct effects on metabolic traits. PLoS Genet 7: e1001324. 21423719 10.1371/journal.pgen.1001324 PMC3053321

[18] Song J, da Costa KA, Fischer LM, Kohlmeier M, Kwock L, et al (2005) Polymorphism of the PEMT gene and susceptibility to nonalcoholic fatty liver disease (NAFLD). FASEB J 19: 1266–1271. 16051693 10.1096/fj.04-3580com PMC1256033

[19] Sookoian S, Castaño G, Gianotti TF, Gemma C, Rosselli MS, et al (2008) Genetic variants in STAT3 are associated with nonalcoholic fatty liver disease. Cytokine 44: 201–206. 18789715 10.1016/j.cyto.2008.08.001

[20] Tokushige K, Hashimoto E, Noto H, Yatsuji S, Taniai M, et al. 2009 Influence of adiponectin gene polymorphisms in Japanese patients with non-alcoholic fatty liver disease. J Gastroenterol 44, 976–98. 10.1007/s00535-009-0085-z 19484180

[21] Li Y, Xing C, Cohen JC, Hobbs HH (2012) Genetic variant in PNPLA3 is associated with nonalcoholic fatty liver disease in China. Hepatology 55: 327–328. 21898508 10.1002/hep.24659 PMC3245325

[22] Fang QC, Jia WP, Gao F, Zhang R, Hu C, et al (2008) Association of variants in APPL1 gene with body fat and its distribution in Chinese patients with type 2 diabetic mellitus. Zhonghua Yi Xue Za Zhi 88(6): 369–373. 18581887

[23] Staiger H, Machicao F, Machann J, Schick F, Kuulasmaa T, et al (2007) Genetic variation within the APPL locus is not associated with metabolic or inflammatory traits in a healthy White population. Diabet Med 24: 817–822. 17490420 10.1111/j.1464-5491.2007.02166.x

[24] Jiang S, Fang Q, Yu W, Zhang R, Hu C, et al (2012) Genetic variations in APPL2 are associated with overweight and obesity in a Chinese population with normal glucose tolerance. BMC Medical Genetics 30: 13–22. 10.1186/1471-2350-13-22 PMC3368742 22462604

[25] Ma XW, Ding S, Ma XD, Gu N, Guo XH (2011) Genetic variability in adapter proteins with APPL1/2 is associated with the risk of coronary artery disease in type 2 diabetes mellitus in Chinese Han population. Chin Med J (Engl). Nov 124(22): 3618–21. 22340213

[26] Chalasani N, Younossi Z, Lavine JE, Diehl AM, Brunt EM, et al (2012) The diagnosis and management of non-alcoholic fatty liver disease: practice guideline by the American Gastroenterological Association, American Association for the Study of Liver Diseases, and American College of Gastroenterology. Gastroenterology 142 (7): 1592–609. 10.1053/j.gastro.2012.04.001 22656328

[27] Zeng MD, Fan JG, Lu LG, Li YM, Chen CW, et al (2008) Chinese National Consensus Workshop on Nonalcoholic Fatty Liver Disease. Guidelines for the diagnosis and treatment of nonalcoholic fatty liver diseases. J Dig Dis 9(2): 108–12. 18419645 10.1111/j.1751-2980.2008.00331.x

[28] Loria P, Adinolfi LE, Bellentani S, Bugianesi E, Grieco A, et al (2010) Practice guidelines for the diagnosis and management of nonalcoholic fatty liver disease. A decalogue from the Italian Association for the Study of the Liver (AISF) Expert Committee Dig Liver Dis 42(4): 272–82. 10.1016/j.dld.2010.01.021 20171943

[29] Saverymuttu SH, Joseph AE, Maxwell JD (1986) Ultrasound scanning in the detection of hepatic fibrosis and steatosis. Br Med J (Clin Res Ed) 292(6512): 13–5. 10.1136/bmj.292.6512.13 PMC1338970 3080046

[30] Bonora E, Targher G, Alberiche M, Bonadonna RC, Saggiani F, et al (2000) Homeostasis model assessment closely mirrors the glucose clamp technique in the assessment of insulin sensitivity. Studies in subjects with various degrees of glucose tolerance and insulin sensitivity. Diabetes Care 23: 57–63. 10857969 10.2337/diacare.23.1.57

[31] Matthews DR, Hosker JP, Rudenski AS, Naylor BA, Treacher DF, et al (1985) Homeostasis model assessment: insulin resistance and beta-cell function from fasting plasma glucose and insulin concentrations in man. Diabetologia 28: 412–9. 3899825 10.1007/BF00280883

[32] Tregouet DA, Escolano S, Tiret L, Mallet A, Golmard JL (2004) A new algorithm for haplotype-based association analysis: the Stochastic-EM algorithm. Ann Hum Genet 68 (Pt 2): 165–77. 10.1046/j.1529-8817.2003.00085.x 15008795

[33] Li YY (2012) Genetic and epigenetic variants influencing the development of nonalcoholic fatty liver disease. World J Gastroenterol 18(45): 6546–51. 23236228 10.3748/wjg.v18.i45.6546 PMC3516206

[34] Choudhury J, Sanyal AJ (2004) Insulin resistance and the pathogenesis of nonalcoholic fatty liver disease. Clin Liver Dis 8: 575–94. 15331065 10.1016/j.cld.2004.04.006

[35] Saito T, Jones CC, Huang S, Czech MP, Pilch PF (2007) The interaction of Akt with APPL1 is required for insulin-stimulated Glut4 translocation. J Biol Chem 282: 32280–32287. 17848569 10.1074/jbc.M704150200

[36] Nechamen CA, Thomas RM, Dias JA (2007) APPL1, APPL2, Akt2 and FOXO1a interact with FSHR in a potential signaling complex. Mol Cell Endocrinol 260–262: 93–99. 10.1016/j.mce.2006.08.014 PMC1782224 17030088

[37] Chial HJ, Wu R, Ustach CV, McPhail LC, Mobley WC, et al (2008) Membrane targeting by APPL1 and APPL2: dynamic scaffolds that oligomerize and bind phosphoinositides. Traffic 9(2): 215–229. 18034774 10.1111/j.1600-0854.2007.00680.x PMC3810297

[38] Puppala J, Siddapuram SP, Akka J, Munshi A (2013) Genetics of nonalcoholic Fatty liver disease: an overview. J Genet Genomics 40(1): 15–22. 23357341 10.1016/j.jgg.2012.12.001

